# The Impact of Emotion and Sex on Fabrication and False Memory Formation

**DOI:** 10.3390/ijerph182212185

**Published:** 2021-11-20

**Authors:** Kamilla Run Johannsdottir, Halldora Bjorg Rafnsdottir, Andri Haukstein Oddsson, Haukur Freyr Gylfason

**Affiliations:** 1 Department of Psychology, Reykjavik University, 102 Reykjavik, Iceland; halldorabjorg@mos.is (H.B.R.); andri@fangelsi.is (A.H.O.); 2 Department of Business, Reykjavik University, 102 Reykjavik, Iceland; haukurgy@ru.is

**Keywords:** false memory formation, fabrication, self-generated errors, sex differences, emotional stimuli

## Abstract

The aim of the present study was to examine how negative emotion and sex affect self-generated errors as in fabrication set-up and later false recognition of those errors. In total, 120 university students volunteered to take part in the study. Participants were assigned at random into two equal sized groups (N = 60) depending on the type of event they received (negative emotional or neutral). We expected that fabrication and false recognition would be enhanced for the emotional event compared to the neutral one. We further hypothesized that both the willingness to fabricate and later false recognition would be enhanced for women compared with men. The results partly confirmed the hypotheses. The results showed that emotional valence (negative) affects both the willingness to fabricate about events that never took place, and the recognition of the fabrication as true at a later point. Women and men were equally likely to fabricate but women were more likely to recognize their fabrication, particularly for the emotional event. The results are discussed in the context of prior work.

## 1. Introduction

Studies on forced fabrication, where misinformation is self-generated by the participant, most commonly use a negatively valanced emotional event as the witnessed event [1,2,3,4]. This is understandable given the overall context of witness interrogation. Emotion, however, particularly negative emotion, seems to play a critical role not only in how events are recalled [5,6] but also in how memory is affected by misinformation [7,8]. Participants are more prone to incorporate misinformation into their memory for negative compared to neutral or positive events [8,9,10,11]. It is unclear, however, what role negative emotions play when the misinformation is self-generated errors as in forced fabrication. Furthermore, studies have shown that women encode and process episodes, as well as, emotional stimuli, differently from men ([12] for overview). For a more comprehensive understanding of fabrication and false memory recognition, it is important to examine potential moderating factors such as emotion and sex differences.

The misinformation effect is when memories are distorted or altered by presenting misleading information after an event [13,14,15]. For example, participants watch a video of an event followed by a statement of another witness including false information about the previously watched event [14,16]. Participants are then questioned about both true events and the false events provided by the misleading information. Fabrication is one manner in which the misinformation effect takes place, where individuals self-generate the misleading information, also termed confabulation [17,18]. In a typical forced fabrication paradigm, participants watch a video of an event and are later asked questions about it that are true (refer to actual events) or false (refer to something that never happened) [1]. Participants are then instructed to answer all questions, guessing if they do not recall the answer (forced fabrication). One week later, the participants complete a true or false recognition test based on their fabricated answers. Several laboratory studies have shown that forcing participants to describe events that never took place increases the likelihood of them later recognizing their fabricated responses as being true [1,2,3,4]. The effect also emerges when participants are asked to completely invent an event on their own [19], especially if the forced fabrication provides a causal explanation for true events [2,20]. The effect is even stronger when participants are strongly encouraged to fabricate (i.e., provided with an option of an “I don’t know” answer), rather than forced to do so [18,21].

The forced fabrication effect seems to be present in all ages; among children and adolescents (elementary students in first and fourth grades, and college students), especially for younger children [1]. When adults are asked to fabricate about an event, participants will later recognize their fabrication as true in approximately 20–40% of the cases [1,2,3,4].

Studies on fabrication and false recognition have commonly used a nine-minute excerpt from the Disney film “Looking for Miracles” as the witnessed event [1,2,3,4,19]. The film clip tells of a boy in a summer camp and is emotional (negative) with a surprise scene of a snake during a boat trip and a quarrel among young campers. Research suggests that misinformation following a negative emotional event has a greater impact on later memory recall or recognition compared to both positive and neutral events [8,9,10,11]. According to the Paradoxical Negative Emotion (PNE) hypothesis, accurately remembering information on negative events is likely to have an evolutionary advantage as it allows the individual to avoid similar situations in the future [7]. This applies also to accepting information on negative events from others. Negative emotional events are therefore likely to be associated with overall enhanced memory of the event, but at the same time to be more prone to distortion (incorporating misinformation) compared to neutral or positive events. Porter et al. [7] had participants recall several public events (some true and some fictional) that varied in their emotional valence (positive and negative). As predicted by the PNE hypothesis, they found that participants had better memory for true negative events compared to true positive events and were also more likely to recall false information related to fictional negative events compared to fictional positive events. The difference between false negative and positive events was considerable, with 90% of the participants recalling at least one false negative event compared to 42% for the positive events. Brainerd et al. [9] found that negative emotional wordlists elicited significantly more false recall compared to neutral wordlists and positive wordlists. In fact, positive wordlists lead to less false recall than neutral wordlists. In a study by Porter, Spencer, and Birt [22], participants saw scenes that were neutral, highly positive, or highly negative (arousal kept constant). Half of the participants received misleading questions, including one that was false. An hour later, participants received questions regarding the scenes seen earlier, that were either correct or referred to the misleading items. The participants who saw the negative emotional scenes were significantly more likely (80%) to recall the false information compared to those who saw the neutral or the positive scenes (40%). Similar results were found by Van Damme and Smets [23], and in Porter et al. [8], misinformation had a greater impact for negative emotional images compared to positive or neutral images.

It is unclear, however, what role negative events may play when misinformation is self-generated as in fabrication studies. Witnessing a negative event may both impact the tendency to fabricate and the recognition of the fabrication as true. Monds et al. [16] unexpectedly found in their study that following a negative event, participants were significantly more likely to self-generate errors (fabricate) compared to a neutral event. Monds et al. set out to test the PNE hypothesis but in addition to supporting the hypothesis, their data also suggested that negative events enhance fabrication. As suggested by Monds et al., it is possible that processing a negative emotional event provides a richer context of personal memories and details, which may lead to more self-generated errors on later recall. This fits with the results of Porter et al. [7], who found that participants tended to take longer time when recalling negative public events (true and false) compared to positive events and include more details in their recall. Furthermore, there is evidence supporting the idea that neutral and negative emotional items are processed differently, with negative emotional items being more likely to activate related material [11,24]. Negative events might therefore encourage more fabrication, as information on something to be avoided is likely to be recognized later as a true event (fitting with the PNE hypothesis). Interestingly, studies have shown that memory organization and encoding of memory differs between men and women, in that women generally store event information with more details [25,26,27] and with greater context, including a more coherent story line, referencing other people and events [28,29]. Therefore, sex in addition to the emotionality of an event may impact fabrication and later false recognition.

Women are known to have superior verbal ability and better performance on various episodic memory tasks compared to men [30,31,32,33,34] for review. Women’s enhanced memory performance compared to men also applies to memory of events and autobiographical memory [35]. Grysman [29] found that women included significantly more internal details in their memory recall of events compared to men and this was true for both short term and longer-term memory retrieval. Similarly, Pillemer et al. [36] found that women have a different memory style compared to men, with women recalling information more in episodes and with more specific information on the recalled events. According to Wang [27], women’s superior memory performance may be linked to the encoding stage. She found that women attended to and encoded far more details about events in their lives compared to men, which later resulted in a richer and more detailed memory of the event. In Grysman [37], 94 participants aged 18–22 were instructed to narrate their memories of events that were up to two years old as well as more recent events (day to one week). The results showed that for both older and more recent events, women’s narratives were more detailed, scored higher on affective measures (more emotional reference), and had more thematic coherence (more story-like). Women also seem to interpret the experienced event more; that is, they included their thoughts, feelings, and reasoning of the experienced event more than men. In general, women tend to refer more to emotional states in their recollection of past experiences [25,28] and are better at recalling emotional information [38,39,40].

The aim of the present study is to specifically examine how negative emotion and sex differences affect self-generated errors as in fabrication set-up and later false recognition of those errors. The altered forced fabrication paradigm by Pezdek et al. [18] was used, where participants are strongly encouraged but not forced to fabricate. This allows us to study both how willing people are to fabricate (self-generate errors) and also examine later false recognition across sex and different events (negative emotional and neutral). Prior studies on fabrication have, for the most part, focused on a negative emotional film clip [1,4,19]. The present study, however, used two film clips, one with a neutral event and the other one with a negative emotional event. Given prior work where negative emotional events impact false memory more than both neutral and positive events, the present study included only neutral and negative emotional film clips.

Following the research outlined above, it is expected that the overall memory for the negative event is enhanced compared to the neutral event, but the negative event will lead to greater fabrication and enhanced recognition of the fabricated answers compared to the neutral event. As previous research has shown, false recognition of previous misinformation is enhanced for negative compared to neutral events and stimuli [7,8,11,23]. Increase in self-generated errors has also been found following a negative compared to a neutral event [16], possibly due to negative emotional events activating a richer context of related information. Accordingly, due to different memory style of women compared to men, with women encoding more contextual information and recalling information more in episodes [26,27,28,29,37], it is expected that women are more willing to fabricate compared to men. Women might also have a better recognition of their fabrication due to their superior episodic memory [31]. The difference between men and women may be particularly apparent following the emotional event given women’s superior emotional judgement [41] and recall of emotional information [38,39]. Furthermore, in accordance with previous research, it is expected that accurate recall is superior in women than in men.

## 2. Method

### 2.1. Participants

To determine the required sample size for participants, a medium effect size of ƒ = 0.30 was chosen with acceptable statistical power (1 – β) = 0.80, which indicated that a sample size of N = 90 was required [42,43]. In total, 120 university students volunteered to take part in the experiment, 66 (55%) females and 54 (45%) males. Their mean age was 25 years (SD = 6.6, range 18–52). Participants were assigned at random to two equal-sized groups (N = 60) depending on the type of event they received. The experiment followed the APA ethical principles and code of conduct and was carried out in accordance with the principles of the Declaration of Helsinki.

### 2.2. Stimuli

Two short video clips, a neutral one and an emotional one, were used in the experiment, both displayed on a 19″ computer screen. For the neutral scene, episode two from season one of the television show “Freaks and Geeks” was used [44]. The scene, approximately 10 minutes long, begins at minute 2:25 and ends at minute 12:06. It displays a neutral scene with high school students discussing a party they were planning to attend later on. For the emotional scene, episode 12 of season one of the television show “Sons of Anarchy” was used [45]. The scene was 11 minutes long, starting at minute 31:01 and ending at minute 42:02. It depicts altercations between gang members, resulting in an innocent woman accidentally being killed by a man who was supposed to murder her husband.

### 2.3. Measures

Two interview sessions were conducted, one immediately following the witnessed events, and one a week later, where participants answered questions on the witnessed events. Eight true questions were posed (referring to events that took place during the scene) and four false questions (referring to events that did not take place) were used to induce false memory development for the participants. One example of a false question for the neutral event is, “What did Lindsay, the main character, give her brother in the hallway?” when in fact, Lindsay did not give her brother anything. At the follow-up interview, a week later, participants answered true or false questions about the same details and events as the questions from the week before. The wording of these questions had been changed to fit the true or false nature of the questions. For the false questions, the participants’ false answers from the week before were incorporated into the questions for each participant depending on what they had answered. For example, if a participant gave the answer “keys” to the question “What did Lindsay, the main character, giver her brother in the hallway?” during the first interview, then the question asked a week later could have been “Lindsay, the main character, gave her brother keys in the hallway–true or false?” For those who did not confabulate to a false question during the first interview (N = 13), a standard question was used at the second interview. The questions on true events were analogous for both scenes, although not the same, because of the different storylines.

The students participated in two slight variations of the study run 8 months apart, where in the second one, a measure of how participants felt (emotion) was conducted before and after the viewing of the video clips. The emotion manipulation check was performed on 80 students and consisted of a five-item mood scale (relaxed, anxious, insecure, happy, and worried) which asked participants to rate how strongly they felt a given emotion at that exact moment, ranging from not at all (1) to very (5). Given the similarity across the two variations, the data sets were combined. Participants who watched the emotional film clip were significantly less relaxed (*p* = 0.022), more anxious (*p* = 0.027), and less happy (*p* = 0.009) compared to the participants who watched the neutral film clip, indicating that the emotional manipulation worked.

### 2.4. Procedure

Participants were informed of how the experiment would be conducted upon arrival. The participants were told that the experiment was a study of how well individuals remember events and details after watching a short video. However, participants were not told about the true nature of the study as it would have threatened the validity of the experiment. After giving their informed consent, participants were tested individually in an interview room and the only other person present during the experiment was the interviewer. All participants were encouraged to give answers to questions they did not know the answers to.

### 2.5. Data Analysis

If participants confabulated an answer to a false question during the first interview, they were given a score of 1, and if they did not answer the question, they were given a score of 0. If participants answered a true event question correctly, they were given a score of 1 and a score of 0 if they answered the question incorrectly. In the second interview, if participants answered the true or false questions; they were given the score of 1 if they said true and a score of 0 if they said false.

## 3. Results

**Figure 1:**
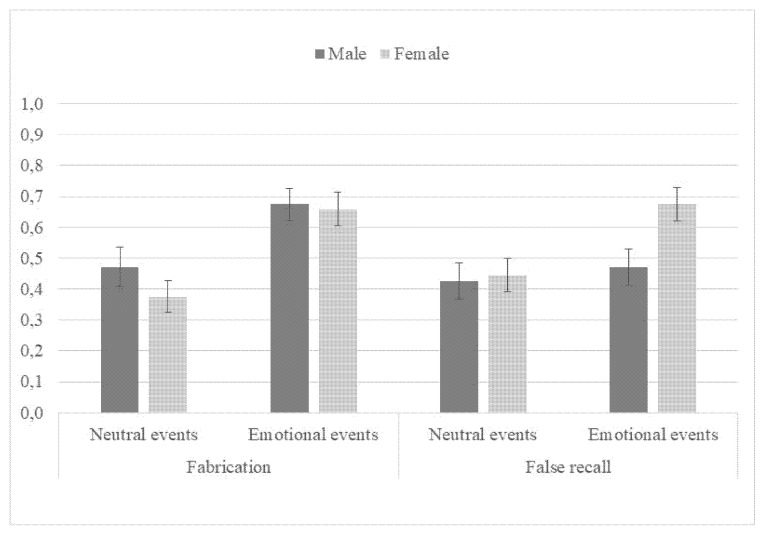
Proportion of fabrication and false recognition (week later) by type of event for men and women. Error bars represent standard error of the mean.

Following the neutral scene, 42% of the false questions; following the emotional scene, participants fabricated answers to 67% (see Figure 1). In total, 11 out of 60 participants did not respond to any of the four false questions following the neutral scene and only 2 out of 60 following the emotional scene.

**Figure 2:**
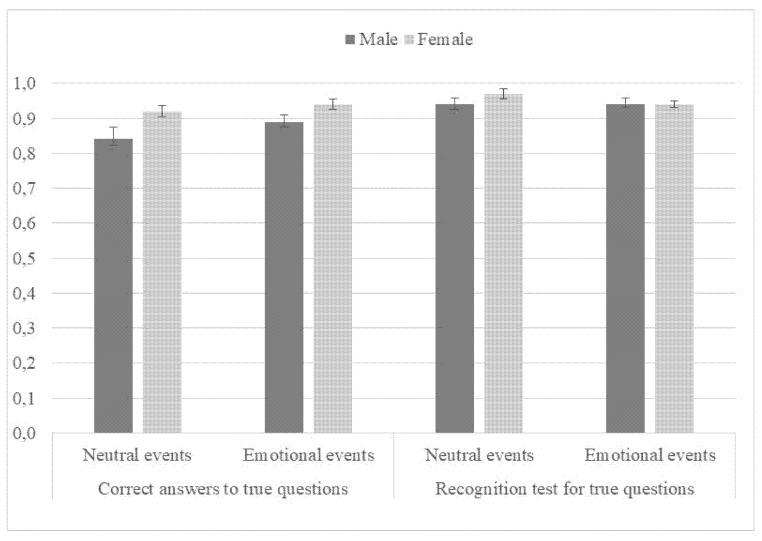
Proportion of correct answers to true questions and for the recognition test for true questions (a week later) by type of event for men and women. Error bars represent standard error of the mean.

For all dependent measures, a separate 2 (type of event: neutral vs. emotional) × 2 (sex: male vs. female) ANOVA was conducted. Participants were more willing to fabricate for emotional compared to neutral false events, *F* (1, 116) = 19.36, *p* < 0.001, *η*^2^*_p_* = 0.14, but recall for true events was similar for emotional and neutral events (see Figure 2), *F* (1, 116) = 2.51, *p* = 0.116, *η*^2^*_p_* = 0.02. Women were equally as willing to fabricate about false events as men, *F* (1, 116) = 1.08, *p* = 0.30, *η*^2^*_p_* = 0.01, but were more accurate in recalling true events, *F* (1, 116) = 10.09, *p* = 0.002, *η*^2^*_p_* = 0.08. Neither interaction terms between sex and type of event were significant (*p* > 0.05).

A week later, participants were contacted to answer true or false questions based on the scene they saw, with those who fabricated answers to the false questions given a false question based on their answer. False recognition was enhanced for the emotional compared to neutral event, *F* (1, 116) = 5.95, *p* = 0.02, *η*^2^*_p_* = 0.05 (see Figure 1). Moreover, false recognition was enhanced for women compared to men, *F* (1, 116) = 3.80, *p* = 0.054, *η*^2^*_p_* = 0.03, although the interaction appeared not statistically significant (*p* > 0.05). However, when controlling for willingness to fabricate, women recognized more fabricated or false answers on average than men, *F* (1, 115) = 10.22, *p* = 0.002, *η*^2^*_p_* = 0.08. Planned comparisons indicated that false assents for women in the emotional group exceeded the false assents for men in the emotional group, *t*(116) = 2.48, *p* = 0.02, *d* = 0.67. Conversely, there was no difference between men and women in the neutral group (*p* = 0.81).

## 4. Discussion

The present study examined the impact of negative emotion and sex differences on self-generated errors (fabrication) and later recognition of those errors. As expected, overall memory was enhanced for negative compared to neutral events and for women compared to men. Furthermore, both fabrication and later false recognition were significantly enhanced for the negative compared to the neutral event. Women were equally as likely to fabricate as men but more likely to later recognize their fabrication as true, particularly for the emotional event.

Previous research has found that misinformation following a negative event is more likely to be later recognized as true compared to neutral or positive event [8,9,10,11]. According to the PNE hypothesis, negative events are both better remembered overall and also more prone to be impacted later by misinformation as there is an evolutionary advantage to remember information related to negative events [7]. The question concerning fabrication and false recognition is whether witnessing a negative event enhances the tendency to fabricate and/or increases the likelihood of the fabrication later being recognized as true compared to witnessing a neutral event. The study followed a typical forced fabrication paradigm [1,4] with the exception that fabrication was strongly encouraged, but not forced [18]. A week later, the participants were asked whether they recognized their generated errors (or standard errors) as true. Misinformation was therefore self-generated.

The results showed that the willingness to fabricate was quite high in the present study, and as expected, significantly higher for the negative emotional event compared to the neutral event. Participants fabricated in 42% of the cases for the neutral event but 67% of the cases for the emotional event, suggesting that when encouraged but not forced to fabricate, participants are more likely to fabricate following a negative emotional event compared to a neutral event. Fabrication as such has not been well studied, but in Pezdek et al. [18], where participants were encouraged but not forced to fabricate, 54% of participants later recognized their fabrication as true after witnessing a negative emotional event. Monds et al. [16] found that following a negative event, participants were significantly more likely to self-generate errors (fabricate) compared to a neutral event. They suggested that this might be due to differential processing of negative and neutral events, with the negative event eliciting richer context of personal memories and details which may lead to more self-generated errors on later recall. The idea of emotional (negative) and neutral material being processed differently has been supported by research [11,24]. Negative emotional material is generally more thematic and interrelated, and therefore perhaps more prone to lead to fabrication [24].

The present results further showed that fabrication enhanced the likelihood of subsequent false recognition, or 44% for the neutral event and 58% for the emotional event. This is considerably higher than previous studies have reported, where false recognition of fabricated answers generally ranges from 20% to 40% for emotional events [1,4]. There was furthermore a significant difference between false recognition of fabricated answers for the emotional event compared to the neutral event, with 58% of the fabrication recognized as true for the emotional event, compared to 44% for the neutral event. Interestingly, the percentage of false recognition during the second interview matched the percentage of fabrication during the first interview for both emotional and neutral events. That is, fabrication was higher for the emotional compared to the neutral event, but participants were no more likely to recognize their fabrication as true following the negative compared to the neutral event.

By allowing both the fabrication and later recognition to vary (i.e., not forcing fabrication), the present study shows that a negative emotional event seems to impact the tendency to fabricate rather than the later recognition of the fabricated answers. This does not fit with the PNE hypothesis. According to the hypothesis, people are more likely to incorporate information provided by others on negative events into their memory compared to positive or neutral events [7]. Accordingly, prior research has shown that misinformation following a negative emotional event is more likely to be later recognized as true compared to neutral or emotional event [8,9,10,11]. Therefore, one might have expected that self-generated errors on negative events would be more likely to be later recognized as true compared to errors on neutral events. This was not the case here. Rather, the present study suggests that the difference between negative emotional and neutral events may lie in how the information is processed and organized at encoding, with emotional events more likely to lead to fabrication (self-generated errors), but the fabrication equally likely to be later recognized as true for both emotional and neutral events.

The results did not find a difference between men and women for fabrication. Various studies have found that women include more details in their recall and encoding of episodes [25,26,27,28,29] and therefore might have been more willing to fabricate. This was not the case here. Prior studies have shown that women encode and organize episodic memories differently from men. For example, they have a better autobiographical memory with more detail and more context [25,26,27,28,29]. Grysman [29] found that women included significantly more internal details in their memory recall of events compared to men. Similarly, Pillemer et al. [36] found that women tend to recall information more in episodes and with more specific information compared to men. This difference in processing of episodes between men and women did, however, not influence their tendency to fabricate. Fabrication therefore seems to be more influenced by emotional valence rather than sex differences. However, women were more likely to recognize their fabrication as true for the emotional event compared to men but the difference was not significant for the neutral event. Women also had better recognition of accurate information for both emotional and neutral events. This fits with prior studies showing that women have superior verbal ability and better performance on various episodic memory tasks compared to men [30,31,32,33,34] for review. Women also tend to be better at recalling emotional information compared to men [38,39]. This might explain why women were more likely to recognize their fabrication or false answers compared to men for the emotional event. Men seemed not to retain their fabrication from interview 1 to interview 2 for the emotional scene. Further research is needed to better understand the role sex differences and emotion play in fabrication and false recognition.

One possible limitation of the experiment concerns the generalizability of the findings. Participants were shown video clips from two different television shows that had not been used before in a study on fabrication and false memory formation. Although participants who watched the emotional film clip were significantly less relaxed, more anxious, and less happy, and participants were significantly more willing to fabricate for emotional events compared to neutral events, it is important to make clear that viewing a traumatizing scene from a television show is different from viewing a real-life event. One way to increase the generalizability would be to vary the valence of real-life events in future studies. Furthermore, it would be of value to the false memory and fabrication literature to study the difference between gender and sex, taking into account that they are distinct concepts, to highlight the important dimensions of variation found in both concepts [46].

## 5. Conclusions

In conclusion, the present study adds important information to the field of fabrication and false memory formation by looking at the impact of emotion and the difference between men and women. Prior work has predominantly used a negative emotional event when studying forced fabrication and false recognition, but studies have shown that the misinformation effect is enhanced for emotional events compared to neutral events. Furthermore, women have been found to be superior at various episodic and emotional memory tasks compared to men. The present study suggests that when misinformation is self-generated, as in fabrication set-up, negative emotional events enhance the tendency to fabricate but do not lead to an enhanced recognition of the fabrication as true at a later point compared to neutral events. Furthermore, women’s superior performance on various episodic memory tasks does not influence their tendency to fabricate, but does enhance their recognition of their fabricated answers compared to men, particularly for emotional events.

## Data Availability

The data presented in this study are available on request from the corresponding author.
